# Diatomite from West Kazakhstan as a Sustainable Raw Material for Borosilicate Glass Production

**DOI:** 10.3390/ma19081503

**Published:** 2026-04-09

**Authors:** Sapura Satayeva, Vera Burakhta, Bekbulat Shakeshev, Firuza Akhmetova, Beksultan Idrisov

**Affiliations:** 1 Industrial and Technological Institute, Zhangir Khan West Kazakhstan Agrarian and Technical University, Uralsk 090009, Kazakhstan; sataeva_safura@mail.ru; 2 Department of Architecture and Construction, West Kazakhstan Innovation and Technological University, Uralsk 090009, Kazakhstan; vburakhta@mail.ru (V.B.); bekshakeshev@mail.ru (B.S.); beksultan.idirisov@mail.ru (B.I.)

**Keywords:** diatomite, quartz sand, chalk, glass batch, heat resistance, chemical resistance, water resistance

## Abstract

This study explores the use of diatomite from West Kazakhstan as a silica-containing raw material for borosilicate glass production. Three glass batches, based on quartz sand, chalk, and diatomite, were synthesized with varying ratios of network-forming and modifying oxides. Partial replacement of quartz sand with diatomite enabled glass melting at 1150 °C, producing a homogeneous melt. Glasses with higher diatomite content showed improved chemical and water resistance; specifically, the sample containing 52% diatomite achieved hydrolytic class III and water resistance class IV (XA = 1.0 cm^3^·g^−1^), whereas quartz-based control samples corresponded to classes IV–V. Heat resistance ranged from 120 to 160 °C depending on composition. These findings demonstrate that amorphous SiO_2_ and active oxides in diatomite promote a stronger three-dimensional glass network, highlighting the potential of locally sourced diatomite as an alternative SiO_2_ source for sustainable, energy-efficient borosilicate glass production.

## 1. Introduction

Glass production remains one of the critical sectors in modern materials science, with borosilicate glasses playing a pivotal role due to their excellent chemical durability, thermal resistance, and mechanical strength, which make them indispensable in laboratory apparatus, chemical processing, and high-temperature applications [1,2,3,4]. Borosilicate glass differs from traditional soda–lime glass by the inclusion of boron trioxide (B_2_O_3_), which significantly lowers the coefficient of thermal expansion and enhances thermal shock resistance [5,6]. The primary glass-forming oxide in these materials is silicon dioxide (SiO_2_), typically derived from high-purity quartz sand, whose quality and composition directly influence glass properties [7,8].

The raw materials used in glass manufacture, such as silica sand, limestone (CaCO_3_), and soda ash (Na_2_CO_3_), determine the glass network structure and performance characteristics [9,10]. While quartz sand remains the predominant source of silica, its limited availability and uneven global distribution challenge the raw material base of the glass industry [11]. Due to these limitations, alternative silica-rich raw materials such as diatomite and other siliceous minerals have attracted increasing research interest [12,13,14].

Diatomite, a siliceous sedimentary rock composed mainly of fossilized diatom shells (opal-A), contains 70–98% SiO_2_ and exhibits a highly porous structure, low density, and large surface area, properties advantageous for multiple industrial applications, including as a silica source in glass and ceramics [15,16]. Diatomite is traditionally used in filtration processes, absorbents, and as a filler in paints and coatings; however, its high amorphous silica content and reactive structure make it a promising alternative raw material for glass production. Compared with crystalline quartz, the amorphous silica in diatomite melts and reacts more readily, which can facilitate batch melting and potentially reduce energy consumption during glass synthesis [17,18]. In addition, the use of diatomite allows for the rational utilization of locally available mineral resources. In the western part of Kazakhstan, significant diatomite deposits exist, including the Zhalpak deposit, which formed in lacustrine sedimentary environments and is characterized by a high content of biogenic amorphous silica and a highly porous microstructure typical of diatomaceous sediments. Utilizing such local resources can reduce dependence on high-purity quartz sand and improve the economic and environmental sustainability of glass production.

Studies have shown that incorporating diatomite and similar high-silica materials like perlite can enable glass formation at lower temperatures compared with conventional batches [19,20]. Additionally, diatomite has been investigated as a substitute raw material for foam and porous glass–ceramic products, demonstrating suitability in thermal insulation materials and sustainable glass variants [21,22]. The ability to derive high-purity silica from diatomite through leaching and alkali extraction has also been demonstrated, further validating its potential as an alternative source for industrial glass manufacture [23]. Beyond silica content, diatomite includes minor oxides (e.g., Fe_2_O_3_, Al_2_O_3_), which can influence melt behavior and structural properties, suggesting opportunities for tailored batch formulations [24,25].

Elemental and phase composition analyses such as XRD and XRF provide critical insights into diatomite suitability by confirming the amorphous silica content and identifying impurity phases that could affect glass properties [26,27]. Likewise, SEM and FTIR characterizations help elucidate surface morphology and functional groups relevant to reactivity in glass melts [28,29]. Research on diatomite from diverse global deposits emphasizes the variability in composition and potential applications, indicating that local raw materials can be effectively utilized in region-specific glass formulations [30,31].

In this study, diatomite from West Kazakhstan, characterized by its unique chemical composition and microstructural features, was investigated as a silica source in borosilicate glass compositions, in combination with conventional raw materials such as quartz sand and chalk. Optimization of the ratio of network-forming and modifying oxides in the glass batches is essential to achieving desirable thermal, chemical, and water resistance properties [32,33]. Previous studies have demonstrated the use of diatomite as a sustainable silica source in glass production, highlighting the importance of raw material characteristics on glass properties and the potential of integrating alternative silica sources to expand the glass raw material base [34,35,36].

Therefore, this study aims to investigate the feasibility of using local diatomite from the West Kazakhstan region as a silica-containing raw material in borosilicate glass production, assessing its influence on glass melt formation, structural characteristics, and performance properties. This work addresses a research gap by evaluating raw, unpurified diatomite as a direct component of glass batches, which remains insufficiently explored in the literature, and demonstrates the potential of locally available resources for sustainable and economically efficient glass manufacturing.

## 2. Materials and Methods

### 2.1. Raw Materials and Batch Composition

Quartz sand from the Gorbunovo deposit (administrative center: Oral, West Kazakhstan Region, Kazakhstan), chalk from the Melovye Gorki deposit (administrative center: Oral, West Kazakhstan Region, Kazakhstan), and diatomite from the Zhalpak deposit (administrative center: Aktobe, West Kazakhstan Region, Kazakhstan) were used as raw materials for glass production. The surface morphologies of quartz sand and chalk were examined prior to batch preparation.

Three glass batch compositions were designed to obtain borosilicate glasses with varying ratios of network-forming and modifying oxides.

Composition I (control), wt.%: quartz sand—42; chalk—8; boric acid (calculated as B_2_O_3_)—23; sodium bicarbonate (calculated as Na_2_O)—18; Al_2_O_3_—7; MgO—2.

Composition II, wt.%: quartz sand—42; diatomite—10; chalk—8; boric acid (B_2_O_3_)—25; sodium bicarbonate (Na_2_O)—15.

Composition III, wt.%: diatomite—52; chalk—8; boric acid (B_2_O_3_)—25; sodium bicarbonate (Na_2_O)—15.

### 2.2. Glass Synthesis

The batch components were thoroughly mixed to ensure homogeneity and placed in 100 mL porcelain crucibles. Melting was carried out in a muffle furnace at 1150 °C with a holding time of 60 min until a homogeneous melt was obtained.

The synthesized glasses differed in the content and ratio of the main oxide components. The resulting samples were subsequently evaluated for thermal stability, chemical resistance, and water resistance.

A constant melting temperature of 1150 °C was applied to all glass compositions in order to ensure identical processing conditions and to enable a direct comparison of the effect of raw material substitution. Due to the presence of boron oxide (B_2_O_3_), which lowers the melting temperature of borosilicate systems, this temperature was sufficient to obtain a homogeneous melt for all investigated compositions. At the same time, it should be noted that the optimal melting temperature may vary depending on the chemical composition of each batch, which can influence melt behavior and the resulting properties of the glass.

### 2.3. Determination of Heat Resistance

Heat resistance was evaluated by repeated heating–cooling cycles. Glass samples were heated in an electric furnace for 15 min at a specified temperature (holding time counted from the moment the set temperature was reached). After heating, samples were rapidly transferred into cold water and held for 30–40 s.

The furnace temperature was increased stepwise by 10 °C in each subsequent cycle until visible signs of degradation (cracking or failure) appeared. The maximum temperature difference sustained without damage was recorded as the heat resistance.

### 2.4. Determination of Chemical Resistance

Chemical resistance was determined by measuring the amount of alkali (Na_2_O equivalent) leached into water according to GOST 10134-2017.

Glass samples were ground to powder, and 2.00 g portions were weighed (±0.01 g) and placed into 100 mL flat-bottom flasks. The powder was rinsed with distilled water, ethanol, or acetone and filtered. Then, 50 mL of distilled water heated to 100 °C was added, and the mixture was refluxed in a boiling water bath for 1 h. After cooling, the solution was transferred to a 250 mL conical flask and titrated with 0.01 N HCl using methyl red as an indicator. Each experiment was performed in triplicate. Results were expressed as milliliters of 0.01 N HCl solution or recalculated as milligrams of Na_2_O. One milliliter of 0.01 N HCl corresponded to 0.31 mg of Na_2_O.

### 2.5. Determination of Water Resistance

Water resistance at 98 °C was determined using Method A according to GOST 10134.1-2017 (for glasses containing alkali metal oxides) [37].

Crushed glass samples were cleaned of dust by repeated rinsing with acetone (or ethanol), dried at 140 ± 2 °C, and cooled in a desiccator. A 2.000 g sample was placed into a 50 cm^3^ volumetric flask, filled with distilled water to the mark, and heated at 98 ± 0.5 °C for 60 min. After cooling, the solution was titrated with 0.01 M HCl using methyl red as an indicator. The water resistance index (X_A_, cm^3^·g^−1^) was calculated using the equation

X_A_ = (V − V_0_)/m

where

V—volume of 0.01 M HCl used for titration of the test solution (cm^3^);

V_0_—volume of 0.01 M HCl used for titration of the control solution (cm^3^);

m—mass of the crushed glass sample (g).

The final X_A_ value was taken as the arithmetic mean of three parallel determinations.

The water resistance class was assigned according to the classification presented in Table 1.

### 2.6. Characterization Techniques

The microstructural, elemental, and phase analyses of the raw materials (quartz sand, chalk, and diatomite) and synthesized borosilicate glasses were carried out using multiple characterization techniques. Scanning electron microscopy (SEM) and energy-dispersive X-ray spectroscopy (EDS) analyses were performed to examine the surface morphology and elemental composition. SEM micrographs were obtained using a high-resolution scanning electron microscope (JEOL JSM-7800F, JEOL Ltd., Tokyo, Japan) with an accelerating voltage of 15–20 kV, a working distance of 10–15 mm, and both secondary electron (SE) and backscattered electron (BSE) detectors. Elemental compositions were determined using an integrated EDS system under the same operating conditions. Powder X-ray diffraction (XRD) measurements were carried out using a diffractometer (Rigaku MiniFlex 600, Rigaku Corp., Tokyo, Japan) equipped with a Cu Kα radiation source (λ = 0.15406 nm), scanned over a 2θ range of 10–80° with a step size of 0.02°, to identify crystalline phases and assess the degree of amorphousness. X-ray fluorescence (XRF) spectroscopy (Table 2, Table 3 and Table 4) was performed using a standard benchtop XRF system (Bruker S8 Tiger, Bruker Corp., Billerica, MA, USA; administrative center: Billerica, MA, USA) to quantify the oxide compositions of raw and heat-treated materials. Fourier-transform infrared (FTIR) spectroscopy was conducted in the 4000–400 cm^−1^ range with a resolution of 4 cm^−1^ (Thermo Scientific Nicolet iS50, Thermo Fisher Scientific, Waltham, MA, USA) to identify functional groups and bonding characteristics. These characterization conditions ensure reproducibility and provide sufficient details for accurate analysis of the materials and glass products.

It should be noted that while EDS and SEM analyses provide valuable information about the local elemental distribution and homogeneity of the raw materials, they cover only a limited surface area (1–100 μm^2^) and may not fully represent the bulk composition. Therefore, bulk chemical composition of the raw materials was additionally determined using X-ray fluorescence (XRF) and other standard chemical methods. This combined approach ensures that the overall composition is accurately characterized, providing reliable data for glass synthesis at both laboratory and industrial scales.

## 3. Results

### 3.1. Characterization of Raw Materials

This subsection presents the results of the microstructural and phase analyses of quartz sand from the Gorbunovo deposit (West Kazakhstan region), which was used as the primary silica-containing raw material in the control glass composition.

**Figure 1:**
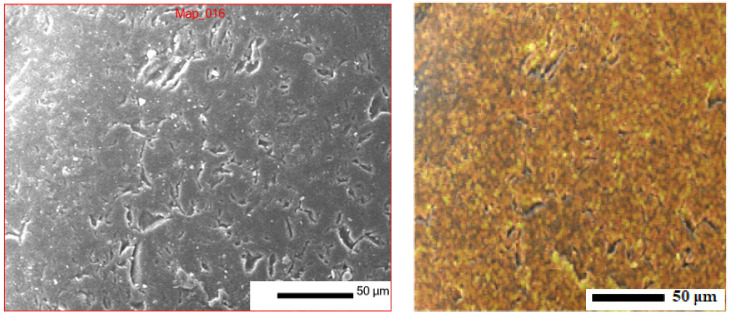
SEM image showing the surface morphology of quartz sand.

Figure 1 shows the SEM image of the quartz sand surface. The quartz grains exhibit a natural morphology with irregular, angular edges and minor traces of surface wear caused by natural mechanical and chemical erosion processes. The surface appears relatively clean, with no significant foreign inclusions, indicating a low level of contamination and good raw material quality.

**Figure 2:**
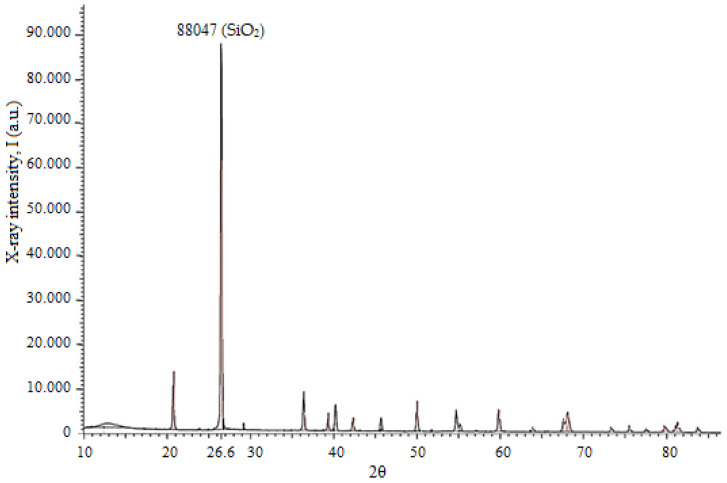
XRD pattern of quartz sand.

The phase composition of the quartz sand was further investigated using X-ray diffraction (XRD) analysis (Figure 2).

The XRD pattern (Figure 2) confirms that the sample is predominantly composed of quartz (SiO_2_). The characteristic diffraction peak observed at 2θ ≈ 26.6° corresponds to the (101) crystallographic plane of quartz. The high intensity and sharpness of this peak indicate a high degree of crystallinity and the predominance of quartz in the sample. Minor low-intensity reflections may correspond to trace secondary phases or impurities; however, their significantly lower intensity confirms the high purity and structural homogeneity of the material.

**Figure 3:**
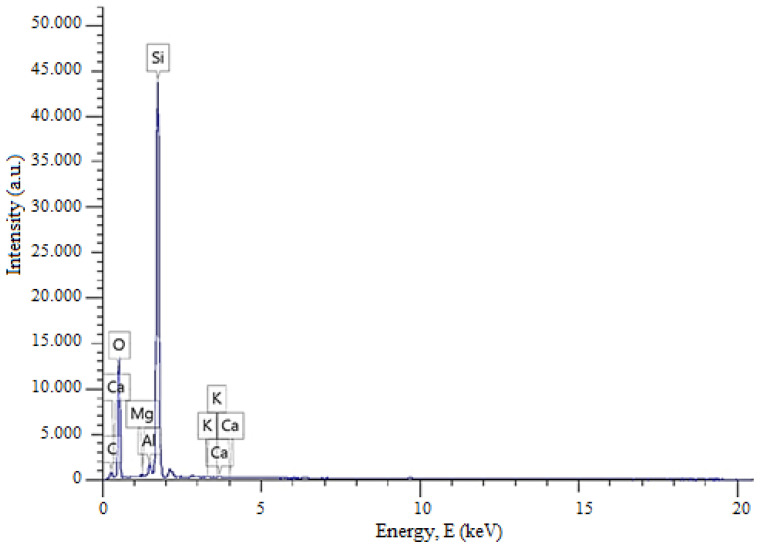
EDS spectrum of quartz sand from the Gorbunovo deposit.

Figure 3 presents the energy-dispersive X-ray spectroscopy (EDS) analysis of quartz sand from the Gorbunovo deposit in the West Kazakhstan region of the Republic of Kazakhstan. The EDS results further confirm the elemental composition and high purity of the sample, consistent with the XRD analysis.

The graph shows the elemental composition of the studied sample, where the peak heights reflect the relative concentrations of the elements, and the peak energies are characteristic of specific chemical elements. In this case, silicon and oxygen exhibit the highest peaks, confirming the quartz nature of the material. The quantitative elemental composition of quartz sand from the Gorbunovo deposit in the West Kazakhstan region of the Republic of Kazakhstan is presented in Table 2.

**Figure 4:**
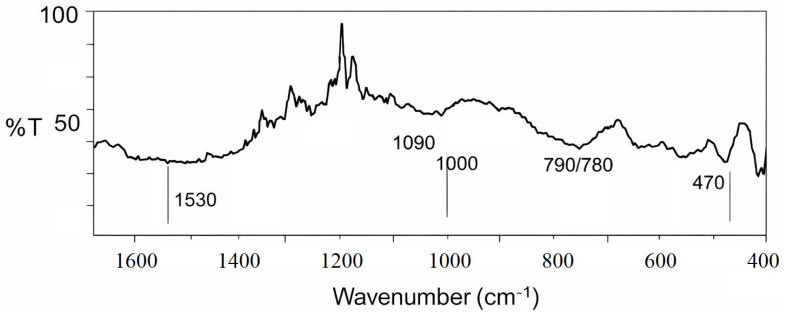
FTIR spectrum of the studied sample in the range of 4000–400 cm^−1^, showing an enlarged view below 1500 cm^−1^.

The XRD and EDS analyses indicate that the studied quartz sand sample predominantly consists of silicon and oxygen, uniformly distributed throughout the structure. Minor elements such as Mg, Al, K, and Ca are present in trace amounts, likely due to localized inclusions of clay minerals or aluminosilicates. This reflects the high purity and structural homogeneity of the material. The relatively high carbon content detected by EDS (Table 2) should not be considered part of the intrinsic composition of quartz sand. The FTIR spectrum of the studied sample (Figure 4), recorded in the range of 4000–400 cm^−1^, is characterized by absorption bands corresponding to Si–O–Si vibrations typical of silica-based materials. For improved visualization and more accurate band assignment, the spectral region below 1500 cm^−1^ is presented in an enlarged view (Figure 4).

The most intense absorption band observed at approximately 1080–1100 cm^−1^ is attributed to the asymmetric stretching vibrations of Si–O–Si bonds, which is characteristic of silica. Additional bands located at around 790–800 cm^−1^ and 460–470 cm^−1^ correspond to the symmetric stretching and bending vibrations of Si–O–Si bonds, respectively. These bands are typical of crystalline silicon dioxide (quartz phase), indicating the presence of a crystalline component in the sample.

No strong or well-defined absorption bands related to carbonate groups (typically observed in the 1400–1500 cm^−1^ region) are present in the spectrum. A weak feature observed near ~1530 cm^−1^ is more likely associated with minor surface contamination or residual organic matter rather than carbonate minerals.

Therefore, the carbon detected by EDS analysis is unlikely to originate from carbonate phases. Instead, it is attributed to loss on ignition (LOI), including surface contamination, residual organic matter (e.g., humic substances), and possible carbon coating applied during SEM–EDS analysis. Accordingly, carbon is not considered a structural component of the material.

These band assignments are consistent with previously reported FTIR spectra of silica and crystalline quartz [38].

In contrast, the FTIR spectra of the synthesized borosilicate glasses exhibit additional absorption bands associated with Si–O–Si and B–O vibrations, confirming the formation of a borosilicate network structure. The absence of additional impurity-related bands indicates the formation of a relatively homogeneous and well-structured glass matrix.

**Figure 5:**
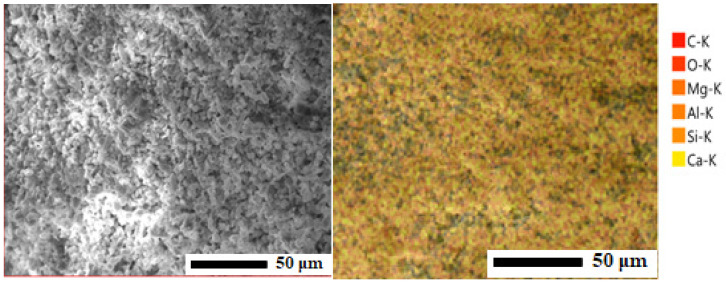
Microstructural image of the chalk surface (SEM).

**Figure 6:**
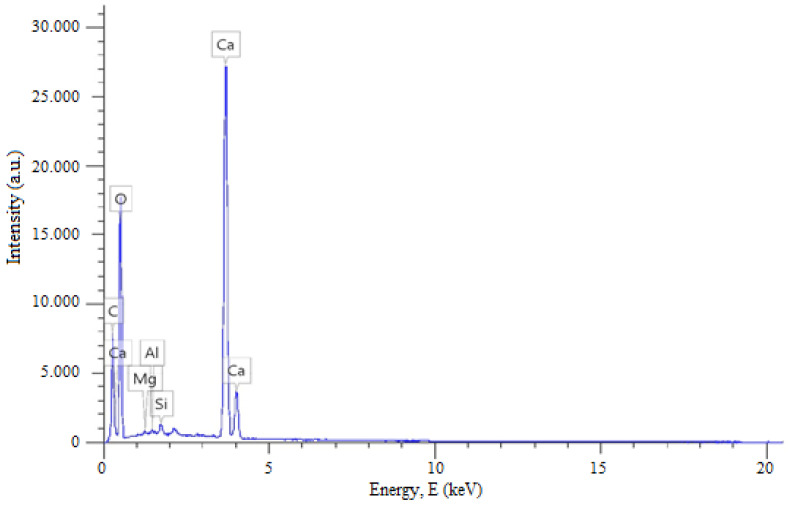
Energy-dispersive X-ray spectroscopy of chalk from the Melovye Gorki deposit.

The structural and elemental composition of chalk from the Melovye Gorki deposit (West Kazakhstan, Republic of Kazakhstan) was studied using scanning electron microscopy (SEM) and X-ray diffraction (XRD) analyses (Figure 5 and Figure 6).

SEM analysis revealed that the chalk has a characteristic fine-grained, granular, and porous surface structure, likely formed by aggregates of individual particles. Variations in grayscale correspond to differences in density: light areas indicate denser or heavier fragments, whereas dark areas indicate lighter or more porous regions.

The elemental composition of the chalk was further determined using energy-dispersive X-ray spectroscopy (EDS) (Figure 6).

The EDS spectrum shows that the chalk is predominantly composed of calcium, oxygen, and carbon, consistent with calcium carbonate (CaCO_3_). The detected carbon (C) signal is attributed to the carbon inherently present in calcite (CaCO_3_), which is the principal mineral phase of the chalk. Minor peaks of silicon, magnesium, and aluminum indicate the presence of natural clay-silicate inclusions. The quantitative elemental composition is summarized in Table 3.

The combined SEM and EDS results confirm that the chalk sample is predominantly composed of calcite (CaCO_3_) with minor natural impurities such as clay and silicate minerals. The fine-grained, porous structure observed by SEM is typical of natural chalk, and the elemental composition supports its natural origin.

As noted, diatomite represents another source of silicon dioxide. Unlike quartz sand, diatomite exhibits a higher reactivity and a porous structure, making it particularly suitable for glass production. Its predominance of amorphous SiO_2_, high purity, and finely dispersed structure facilitate melting reactions, reduce glass melting temperatures, and lower energy costs. Additionally, its natural origin and local availability make diatomite an economically viable and environmentally friendly raw material for the glass industry.

**Figure 7:**
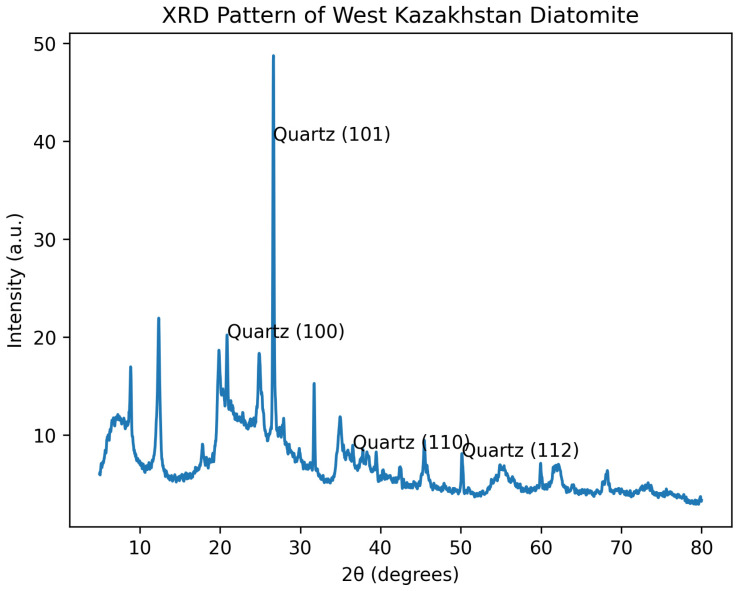
XRD pattern of West Kazakhstan diatomite with a broad amorphous halo (2θ ≈ 20–25°) and weak crystalline reflections of quartz.

The X-ray diffraction (XRD) pattern of diatomite from West Kazakhstan is shown in Figure 7. The diffractogram exhibits a broad diffuse halo in the range of 2θ ≈ 20–25°, which is characteristic of amorphous silica and confirms that the dominant phase is biogenic opal (SiO_2_·nH_2_O).

In addition to the amorphous phase, several distinct diffraction peaks are observed, indicating the presence of minor crystalline components. The most intense peak at 2θ ≈ 26.6° is indexed to α-quartz (SiO_2_), corresponding to the (101) crystallographic plane (ICDD PDF No. 46-1045).

Additional reflections at approximately 2θ ≈ 20.9°, 36.5°, and 50.1° are also attributed to α-quartz, corresponding to the (100), (110), and (112) planes, respectively.

The presence of these reflections confirms that quartz is the main crystalline impurity in the studied diatomite. Minor low-intensity peaks may also be related to trace silicate or clay mineral phases typically associated with natural sedimentary deposits.

The dominance of the amorphous halo over crystalline peaks suggests that the material predominantly consists of biogenic amorphous silica with a low degree of crystallinity. Such structural characteristics are typical for natural diatomite and are highly beneficial for glass production, since amorphous silica demonstrates higher reactivity compared to crystalline quartz. The presence of minor crystalline quartz does not significantly affect the suitability of the material for borosilicate glass synthesis; however, it may slightly increase the melting temperature compared to fully amorphous silica. Overall, the XRD results confirm that West Kazakhstan diatomite is mainly composed of amorphous SiO_2_ with small amounts of crystalline phases, making it a promising sustainable raw material for borosilicate glass production.

The XRF data are presented as elemental equivalents recalculated from oxide compositions. The results are summarized in Table 4. Thermal treatment induces notable changes in the elemental composition of diatomite, primarily due to the removal of physically and structurally bound water and the redistribution of oxide phases.

As shown in Table 4, thermal treatment results in an increase in the mass fraction of silicon from 32.283% to 37.924%, indicating silica enrichment of the material. The iron content also increases significantly, from 26.008% to 35.665%, while potassium, titanium, and calcium show moderate increases. These changes suggest activation of glass-forming and modifying oxides during heating, which is beneficial for melt formation in glass production.

In contrast, a substantial decrease in aluminum content is observed, from 24.400% to 4.364%, along with reductions in copper, arsenic, and manganese. This behavior may be attributed to partial volatilization, phase transformation, or redistribution of these elements during thermal modification. The observed changes in elemental composition confirm the structural transformation of diatomite during heat treatment and indicate an increase in its reactivity and glass-forming potential, supporting its use as an effective alternative silica-containing raw material in glass batch formulations.

### 3.2. Characterization of Glass Products

**Figure 8:**
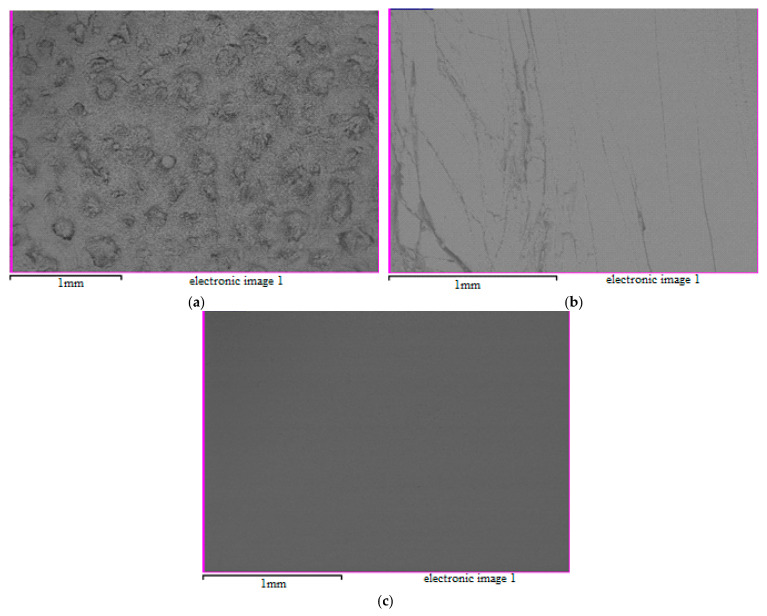
SEM micrographs of borosilicate glasses synthesized from: (**a**) Composition I; (**b**) Composition II; (**c**) Composition III.

The microstructure of the synthesized borosilicate glasses was investigated using scanning electron microscopy (SEM), and the results are presented in Figure 8.

As shown in Figure 8a, the glass corresponding to Composition I (control sample) exhibits a relatively dense amorphous structure; however, slight microstructural heterogeneities can be observed. Although the surface appears generally smooth, minor contrast variations suggest incomplete homogenization of the melt. This may be attributed to the limited reactivity of quartz sand, which typically requires higher temperatures and longer melting durations for complete dissolution.

In contrast, the SEM image of Composition II (Figure 8b), which contains both quartz sand and diatomite, demonstrates a noticeably improved microstructure. The glass appears more uniform, with reduced contrast variations and fewer localized inhomogeneities. The partial replacement of quartz with diatomite enhances silica dissolution due to its amorphous nature, high porosity, and large specific surface area. As a result, more efficient interaction between silica and calcium-containing components is achieved, promoting better melt homogenization and formation of a more uniform silicate network.

A further improvement is observed in Composition III (Figure 8c), where quartz sand is completely replaced by diatomite. The SEM micrograph reveals a highly homogeneous and dense amorphous structure with a smooth and uniform morphology. No visible crystalline inclusions, sharp boundaries, or phase separations are detected. This indicates that the use of diatomite as the primary silica source significantly enhances melt reactivity and facilitates complete glass network formation under the same processing conditions.

The observed differences in microstructure can be explained by the physicochemical properties of diatomite from West Kazakhstan, which is rich in amorphous silica and characterized by a highly porous structure. These features accelerate silica dissolution and improve mass transfer during melting, resulting in a more uniform distribution of network-forming and modifying oxides.

Overall, the comparative analysis of SEM images clearly demonstrates that increasing the proportion of diatomite (from Composition I to III) leads to progressive improvement in microstructural uniformity. The incorporation of diatomite suppresses microstructural defects, enhances melt homogenization, and promotes the formation of a consistent borosilicate glass network. These results strongly confirm that diatomite from West Kazakhstan is a highly effective and sustainable alternative silica source for borosilicate glass production, offering improved structural quality compared to conventional quartz-based systems.

To further correlate the observed microstructural features with compositional variations, the combined SEM and EDS results reveal a clear structure–property relationship. The progressive incorporation of diatomite leads to improved microstructural homogeneity, as evidenced by the transition from heterogeneous regions in Composition I to a dense and uniform amorphous matrix in Composition III. This structural refinement is directly associated with the increased presence of alkali and alkaline earth elements (Na, K, Mg) identified by EDS, which act as network modifiers. These modifiers reduce melt viscosity and enhance mass transfer during melting, promoting more complete homogenization and formation of a stable silicate network. Consequently, the improved structural uniformity is expected to result in enhanced physicochemical properties, including better thermal stability, chemical durability, and reduced defect density. Thus, the use of diatomite not only alters the composition but also positively influences the structure and performance of the resulting glass materials.

**Figure 9:**
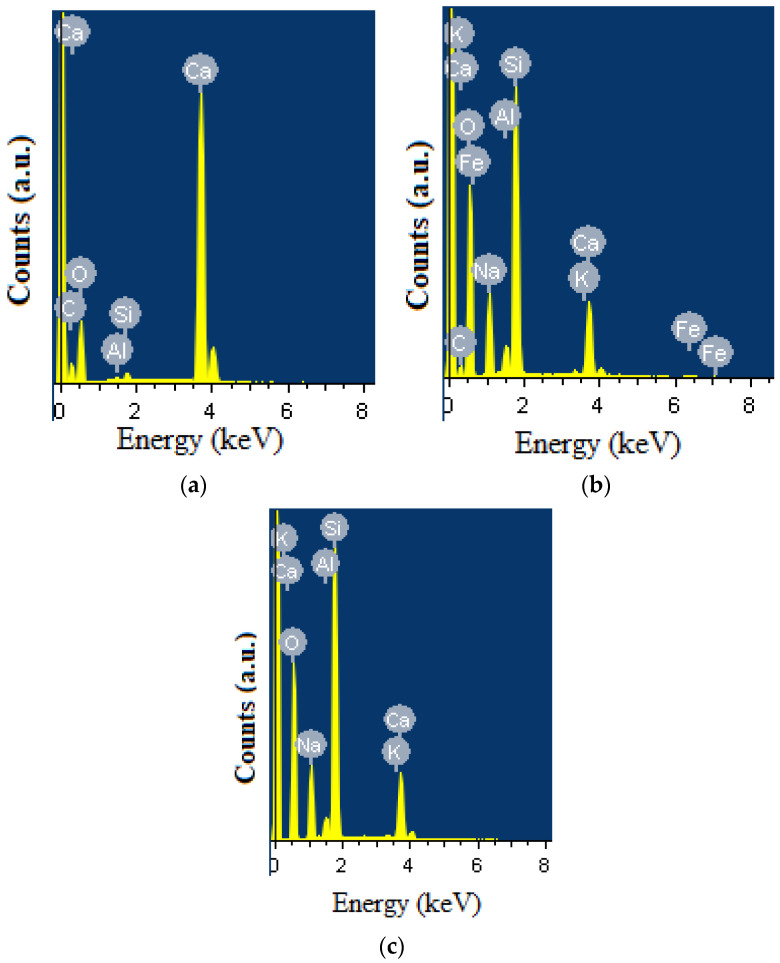
Energy-dispersive X-ray spectroscopy (EDS) spectra of borosilicate glasses synthesized from different raw material compositions: (**a**) Composition I (quartz sand + chalk), (**b**) Composition II (quartz sand + diatomite), and (**c**) Composition III (diatomite as the primary silica source).

To further verify these compositional effects, energy-dispersive X-ray spectroscopy (EDS) was employed to analyze and compare the elemental composition of borosilicate glass samples synthesized from different raw material compositions. The corresponding spectra are presented in Figure 9.

As shown in Figure 9a, the EDS spectrum of Composition I (control sample), prepared using quartz sand from the Gorbunovo deposit and chalk from the Melovye Gorki deposit, is dominated by oxygen (O), silicon (Si), and calcium (Ca). These elements represent the primary constituents of the silicate glass network and the calcium source introduced by chalk. The pronounced Ca peak confirms the contribution of calcium-containing fluxes, while the strong Si and O signals correspond to the silicate framework. A minor carbon (C) signal is also detected, which is most likely related to surface contamination or sample preparation rather than the intrinsic composition of the glass.

The peaks initially attributed to aluminum (Al) were carefully re-evaluated. Their positions do not fully correspond to the characteristic Al Kα energy (~1.49 keV), indicating that the previous assignment may not be accurate. In particular, the peak observed near ~1.7 keV is more consistent with Si, while the feature around ~2.1 keV may arise from peak overlap or background effects. Therefore, the presence of Al in Composition I cannot be conclusively confirmed from the current EDS data and, if present, is likely at trace levels.

In Composition II (Figure 9b), where diatomite is introduced alongside quartz sand, the elemental composition becomes more diverse. In addition to the major elements (O, Si, Ca), peaks corresponding to sodium (Na), magnesium (Mg), potassium (K), iron (Fe), and titanium (Ti) are observed. These elements originate from the natural mineral composition of diatomite and indicate the incorporation of additional oxides into the glass structure. Their presence suggests partial modification of the glass network due to the introduction of an alternative silica source.

A more pronounced effect is observed in Composition III (Figure 9c), where diatomite completely replaces quartz sand as the primary silica source. The EDS spectrum shows relatively higher intensities of alkali (Na, K) and alkaline earth (Mg) elements compared to Composition II, confirming their increased incorporation into the glass matrix. At the same time, the dominant Si and O peaks remain, indicating preservation of the silicate network structure. The presence of Fe and Ti, although in minor amounts, further reflects the natural impurity profile of diatomite.

The incorporation of alkali (Na, K) and alkaline earth (Mg) elements plays an important role in glass formation, as these oxides act as network modifiers. Their presence reduces the viscosity and melting temperature of the glass melt, thereby enhancing melt fluidity and homogenization. Furthermore, trace transition metals such as Fe and Ti may influence the optical properties and chemical durability of the resulting glass, even at low concentrations.

Overall, the comparative EDS analysis demonstrates that increasing the proportion of diatomite (from Composition I to III) leads to a progressive enrichment of the glass composition with modifying oxides. This modification contributes to improved melting behavior and processing efficiency. The results confirm that diatomite from West Kazakhstan serves not only as a silica source but also as a multifunctional raw material that enhances the physicochemical properties of borosilicate glass. These compositional changes are expected to influence the structural ordering within the glass, which can be further investigated using XRD analysis.

X-ray diffraction (XRD) patterns of all synthesized glass samples were recorded to evaluate the presence of crystalline phases. The obtained diffraction patterns exhibit broad halos typical of amorphous materials, without sharp peaks corresponding to crystalline phases. Minor features observed in some samples were compared with standard reference patterns from the International Centre for Diffraction Data (ICDD) database; however, no matches with known crystalline phases were identified. This confirms that the glasses are fully amorphous. The results indicate that the selected synthesis conditions (temperature and duration) are sufficient to ensure complete melting of the raw materials and the formation of homogeneous glass without crystallization or phase separation.

**Figure 10:**
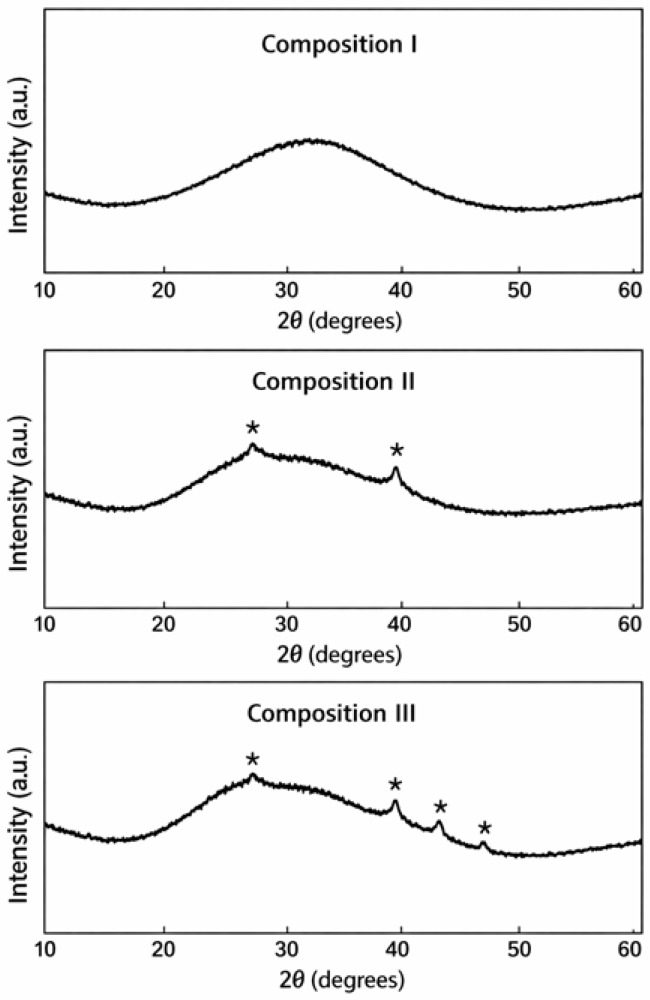
XRD patterns of Compositions I, II, and III showing weak diffraction peaks (*).

The XRD patterns presented in Figure 10 were analyzed to identify the crystalline phases corresponding to the weak diffraction peaks marked with (*).

For Composition I, the XRD pattern is dominated by a broad diffuse halo centered at 2θ ≈ 30–32°, which is characteristic of a predominantly amorphous borosilicate glass structure. No distinct sharp diffraction peaks are observed, indicating that crystalline phases, if present, exist only in trace amounts. This suggests that silica and other components are largely dissolved within the glass network.

In Composition II, a weak diffraction peak at approximately 2θ ≈ 26.6° is attributed to cristobalite (SiO_2_) (ICDD PDF No. 00-082-0512). An additional feature near 2θ ≈ 39–40° may correspond to wollastonite (CaSiO_3_) (ICDD PDF No. 00-043-1460), formed through the interaction between SiO_2_ and CaO during melting. The low intensity of these peaks indicates that these crystalline phases are present only in minor quantities.

In Composition III, similar weak diffraction peaks are observed within the 2θ ≈ 27–48° range, indicating a slightly increased degree of structural ordering. The peak near 26.6° again corresponds to cristobalite (SiO_2_) (ICDD PDF No. 00-082-0512), suggesting partial crystallization of amorphous silica during cooling. Peaks around 39–41° may correspond to wollastonite (CaSiO_3_) (ICDD PDF No. 00-043-1460), and additional weak reflections in the 44–46° range are associated with Fe- and Ti-containing oxides, such as hematite (Fe_2_O_3_) or rutile (TiO_2_), originating from natural impurities in diatomite.

Despite these minor crystalline phases, all samples remain predominantly amorphous, as evidenced by the dominant broad halo. This confirms that the fundamental borosilicate glass network is preserved, and that diatomite from West Kazakhstan can be effectively used as an alternative silica source without compromising the structural integrity of the glass. Overall, increasing the proportion of diatomite slightly promotes the formation of secondary crystalline phases, but the amorphous nature of the glass is retained.

### 3.3. Thermal, Chemical, and Water Resistance of Glass

The thermal stability, chemical resistance, and water resistance of the synthesized borosilicate glass samples were evaluated to assess the influence of glass batch composition on material properties.

**Figure 11:**
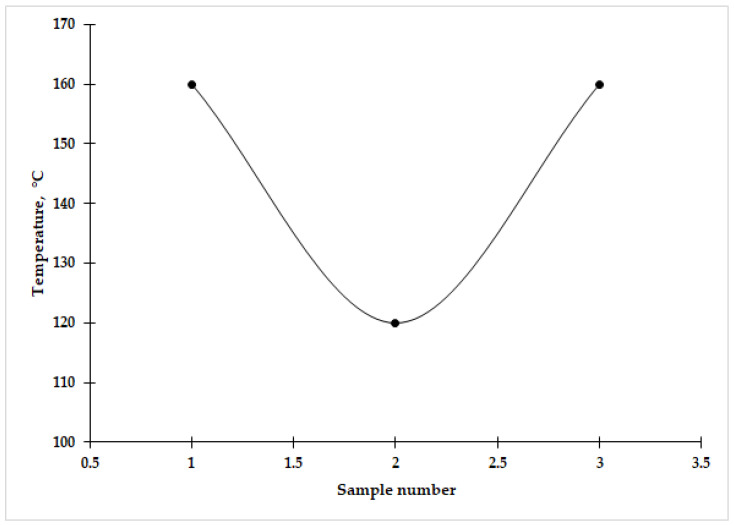
Results of determination of heat resistance of borosilicate glass.

The results of the heat resistance tests are presented in Figure 11. Glass compositions I and III exhibit the highest thermal stability, withstanding temperature differences up to 160 °C without visible damage. In contrast, composition II shows lower thermal resistance, approximately 120 °C, likely due to variations in the ratio of network-forming to modifying oxides, which increase the coefficient of thermal expansion. The higher thermal stability of samples I and III indicates the formation of a stronger and more thermally stable three-dimensional network.

The chemical resistance of the glass samples was assessed by measuring the amount of alkali (Na_2_O) released into water, following GOST 10134-2017. The results are summarized in Table 5. Samples I and II are classified as hydrolytic class IV, indicating relatively low chemical resistance, while sample III shows higher resistance (class III), as evidenced by lower HCl consumption and minimal Na_2_O release.

Water resistance tests at 98 °C confirmed similar trends (Table 6). Samples I and II belong to water resistance class V, with higher XA values indicating more intense leaching. Sample III exhibited the lowest XA value, corresponding to minimal component leaching and the highest water resistance.

To ensure consistency in the chemical and water resistance tests, all synthesized glass samples were ground and sieved to a uniform particle size of 100–150 µm. This standardization eliminates surface area as a variable, allowing differences in resistance to be attributed primarily to variations in chemical composition and network structure.

The differences observed in the properties of the synthesized glasses are related not only to the use of diatomite as a silica source but also to variations in the overall chemical composition. Since the batch compositions differ in the ratio of network-forming and modifying oxides, the resulting glasses have different oxide contents, which significantly influence thermal, chemical, and water resistance. Therefore, the improved properties of diatomite-containing glasses are due to the combined effect of amorphous silica, higher reactivity, and its influence on the glass network structure, rather than solely the presence of diatomite.

The diatomite used in our study contains a significant amount of iron oxide, imparting a greenish tint to the resulting glasses. In contrast, glasses prepared from quartz sand, which contain negligible iron, appear colorless. While this work focuses on thermal and mechanical properties, this note clarifies differences in optical appearance.

The glass transition temperatures (Tg) of the synthesized borosilicate glasses were measured using differential scanning calorimetry (DSC), providing insight into structural variations among compositions. Tg values indicate the temperature at which the glass network softens, reflecting the connectivity and rigidity of the silicate–borate framework. Additionally, the coefficients of thermal expansion (CTE) were determined, showing that diatomite-containing glasses exhibit slightly higher CTE values due to minor fluxing oxides and the influence of amorphous silica on the network. Complementary FTIR spectra confirm the formation of fully connected silicate–borate networks, correlating structural features with thermal behavior.

All synthesized glasses were visually inspected and analyzed using X-ray diffraction (XRD) to verify the formation of a fully amorphous structure. No crystalline phases or phase separation were detected, and all glasses appeared homogeneous macroscopically. As noted, diatomite-containing glasses exhibited a slight greenish tint, while quartz-based glasses were colorless and transparent. No significant opacity or inhomogeneity was observed, confirming that the applied processing parameters allowed the formation of uniform borosilicate glasses.

## 4. Conclusions

Comprehensive structural and physicochemical analyses confirmed the suitability of local mineral raw materials for borosilicate glass production. Quartz sand from the Gorbunovo deposit exhibited high crystallinity and purity, while chalk from the Melovye Gorki deposit consisted predominantly of calcite (CaCO_3_) with minor natural impurities. Diatomite from West Kazakhstan was mainly amorphous SiO_2_ with minor crystalline inclusions, providing higher chemical reactivity than crystalline quartz. Thermal treatment of diatomite enhanced silica content and activated glass-forming and modifying oxides, increasing its reactivity and glass-forming potential. Partial replacement of quartz sand with diatomite allowed glass melting at 1150 °C and improved melt homogenization, as evidenced by SEM observations of dense and uniform amorphous microstructures.

The physicochemical properties of the synthesized glasses strongly depended on batch composition. The sample containing 52% diatomite (Sample III) exhibited the best overall performance, achieving hydrolytic class III chemical resistance and class IV water resistance (XA = 1.0 cm^3^·g^−1^), whereas glasses without diatomite corresponded to lower resistance classes (IV–V). Thermal resistance ranged from 120 to 160 °C, with the highest stability observed in compositions with optimized ratios of network-forming and modifying oxides. The improved properties are attributed to the formation of a stronger three-dimensional silicate network, facilitated by amorphous silica and active oxides in diatomite.

Overall, the study demonstrates that locally sourced diatomite can effectively replace quartz sand in borosilicate glass production, enabling lower melting temperatures, enhanced structural homogeneity, improved chemical durability, and increased energy efficiency. These findings support the development of sustainable, resource-efficient glass manufacturing technologies.

## Figures and Tables

**Table 1:** Determination of water resistance class by consumption of 0.01 M HCl solution during titration.

Consumption of 0.01 M HCl Solution During Titration, cm^3^	Water Resistance Class
up to 0.10	I
0.10–0.20	II
0.20–0.85	III
0.85–2.00	IV
2.00–3.50	V

**Table 2:** Elemental composition of quartz sand from the Gorbunovo deposit in the West Kazakhstan region of the Republic of Kazakhstan.

Element	Mass Fraction (%)	Atomic Fraction (%)
C	12.36 ± 0.11	19.57 ± 0.18
O	41.31 ± 0.15	49.11 ± 0.17
Mg	0.18 ± 0.01	0.14 ± 0.01
Al	1.15 ± 0.02	0.81 ± 0.01
Si	44.50 ± 0.11	30.13 ± 0.07
K	0.23 ± 0.01	0.11 ± 0.01
Ca	0.27 ± 0.02	0.13 ± 0.01

**Table 3:** Elemental composition of chalk in the West Kazakhstan region of the Republic of Kazakhstan.

Element	Mass Fraction (%)	Atomic Fraction (%)
C	15.42 ± 0.04	23.53 ± 0.06
O	54.73 ± 0.17	62.68 ± 0.19
Mg	0.17 ± 0.01	0.13 ± 0.01
Al	0.12 ± 0.01	0.08 ± 0.01
Si	0.35 ± 0.01	0.23 ± 0.01
Ca	29.20 ± 0.07	13.35 ± 0.03

**Table 4:** Oxide composition determined by XRF of natural and heat-treated diatomites.

Element	Weight Composition of Diatomite, %	
	Natural	Thermally Modified
Fe	26.008	35.665
Ti	1.582	2.933
K	4.589	7.663
Ca	0.382	0.548
Cr	0.108	0.123
Al	24.400	4.364
Si	32.283	37.924
Cl	1.081	1.308
Zn	0.380	0.555
Cu	0.228	0.061
As	0.237	0.163
Rb	0.077	0.348
Sr	0.044	0.306
Mn	0.055	0.036

**Table 5:** Results of determination of chemical resistance of manufactured glasses.

Sample Number	Amount of HCl Used for Titration, mL	Amount of Na_2_O, mg	Hydrolytic Class for Chemical Resistance (GOST 10134-2017)
I	6.3	1.96	IV
II	4.7	1.46	IV
III	2.5	0.78	III

**Table 6:** Results of determining the water resistance of glass samples at 98 °C.

Sample Number	V_0_, mL HCl	V, mL HCl	X_A_, cm^3^·g^−1^	Water Resistance Class (GOST 10134-2017)
I	0.5	6.3	3.8	V
II		4.7	2.1	V
III		2.5	1.0	IV

## Data Availability

The original contributions presented in this study are included in the article. Further inquiries can be directed to the corresponding author.
